# Experimental murine acute lung injury induces increase of pulmonary TIE2-expressing macrophages

**DOI:** 10.1186/s12950-018-0188-5

**Published:** 2018-06-14

**Authors:** Heidi Ehrentraut, Christina Weisheit, Marcel Scheck, Stilla Frede, Tobias Hilbert

**Affiliations:** Department of Anesthesiology and Intensive Care Medicine, University Hospital Bonn, Sigmund-Freud-Str. 25, 53127 Bonn, Germany

**Keywords:** Acute lung injury, Inflammation, TIE2, Monocytes, Macrophages, Angiopoietin

## Abstract

**Background:**

Breakdown of the alveolo-capillary wall is pathognomonic for Acute Lung Injury (ALI). Angiopoietins, vascular-specific growth factors, are linked to endothelial barrier dysfunction, and elevated Angiopoietin-2 (ANG2) levels are associated with poor outcome of ALI patients. Specialized immune cells, referred to as ‘TIE2-expressing monocytes and macrophages’ (TEM), were shown to specifically respond to ANG2 binding. However, their involvement in acute inflammatory processes is so far completely undescribed. Thus, our aim was to assess the dynamics of TEMs in a murine model of ALI.

**Results:**

Intratracheal instillation of LPS induced a robust pulmonary pro-inflammatory response with endothelial barrier dysfunction and significantly enhanced ANG2 expression. The percentage number of TEMs, assessed by FACS analysis, was more than trebled compared to controls, with TEM count in lungs reaching more than 40% of all macrophages. Such distinct dynamic was absent in all other analyzed compartments (alveolar space, spleen, blood). Incubation of the monocytic cell line THP-1 with LPS or TNF-α resulted in a dose-dependent, significant upregulation of TIE2, suggesting that not recruitment from extra-pulmonary compartments but TIE2 upregulation in resident macrophages accounts for increased lung TEM frequencies.

**Conclusions:**

For the first time, our data provide evidence that the activity of TEMs changes at sites of acute inflammation.

**Electronic supplementary material:**

The online version of this article (10.1186/s12950-018-0188-5) contains supplementary material, which is available to authorized users.

## Background

Acute Lung Injury (ALI) and Acute Respiratory Distress Syndrome (ARDS) are characterized by an overwhelming host response to severe viral or bacterial infection, aspiration, or severe shock. They lead to heavily increased permeability for proteins and fluids across the alveolo-capillary wall, accompanied by a massive transmigration of leukocytes [1]. Due to the resulting alveolar as well as interstitial edema and infiltration, patients suffering from ALI/ARDS rapidly develop acute respiratory failure with arterial hypoxemia and impaired carbon dioxide elimination. With incidences of up to 80/100.000 person-years and a reported mortality between 40 and 50% even in times of modern therapy strategies (lung-protective ventilation, extracorporeal membrane oxygenation), ALI/ARDS represents a severe medical and socio-economic burden [2–4].

Angiopoietins are vascular-specific growth factors that are directly linked to the barrier function of the endothelium [5]. Since angiopoietin (ANG) 2 acts as competitive antagonist for ANG1 at the specific endothelial receptor TIE2, excessive ANG2-mediated blockade of TIE2 has been demonstrated to induce breakdown of the vascular barrier in vitro, in animal models as well as clinical settings [6–8]. A recent study reports about the association of elevated ANG2 plasma levels with fluid balance and poor clinical outcome of patients suffering from ALI [9]. Direct interaction of ANG2 with TIE2 is thought to be the primary mechanism [7].

In recent years, studies in the field of tumor biology have provided evidence that, besides endothelial cells (ECs), another cell population may specifically respond to angiopoietin binding. Due to their surface characteristics (TIE2^+^, CD45^+^, CD11b^+^), they were referred to as ‘TIE2-expressing monocytes or macrophages’ (TEM) [10]. It has been demonstrated that, in response to hypoxia-induced overexpression of chemoattractive ANG2 in the center of a growing, solid tumor, TEMs migrate into the neoplastic tissue. There, they allow for further tumor growth by exerting distinct pro-angiogenic properties [11]. In addition, they facilitate invasive expansion as well as metastatic spread by increased secretion of vascular leakage-related or matrix-degrading proteins and immunomodulating cytokines [12].

There are very few reports on an involvement of TEMs in pathologies other than cancer, and despite obvious pathophysiologic similarities between tumor biology and inflammation, a possible contribution of TEMs to acute inflammatory processes is so far completely undescribed [13]. Thus, our aim was to assess the dynamics of TEMs in a murine model of ALI. We hypothesized that ALI induction affects the frequency of TEMs in the lungs. Our results may provide first evidence for an alternative involvement of the angiopoietin/TIE receptor system in the development of ALI/ARDS.

## Methods

### Materials and reagents

PBS (pH 7.4) was purchased from Life Technologies (Carlsbad, CA, USA). Cell culture plastics (10-cm dishes, 6- and 96-well flat-bottom plates) were obtained from Greiner Bio-One (Frickenhausen, Germany). The TLR4 ligand lipopolysaccharide (LPS; *Escherichia coli* O111:B4) was purchased from Sigma-Aldrich (St. Louis, MO, USA). Recombinant human TNF-α (cat. Code 210-TA) was obtained from R&D Systems (Wiesbaden-Nordenstadt, Germany). 4 M guanidinthiocyanat was purchased from Roth (Karlsruhe, Germany) and TRIzol™ reagent from Life Technologies (Carlsbad, CA, USA). Accutase™ solution, chloroform, isopropanol and ethanol were obtained from Sigma-Aldrich (St. Louis, MO, USA). Phorbol 12-myristate 13-acetate (PMA, cat. Code P8139), thrombin from human plasma (cat. Code T7009) and histamine (cat. Code H7125) were purchased from Sigma-Aldrich as well. Reagents for Fluorescent Activated Cell Sorting (FACS) were purchased from Miltenyi Biotec (Bergisch Gladbach, Germany), eBioscience (Waltham, Massachusetts, USA) and BD Biosciences (San Jose, CA, USA), respectively. Assay buffer for FACS was prepared by adding 0.5% FBS and 2 mM EDTA (0.5 M, pH 8.0; Merck KGaA, Darmstadt, Germany) to PBS.

### Cell culture and stimulation

Cryopreserved pooled human pulmonary microvascular endothelial cells (HMVECs) and the recommended cell culture medium (ECGM-MV (endothelial cell basal medium with cell-specific supplements)) were purchased from PromoCell (Heidelberg, Germany). Cells of less than 6 passages were cultured in medium in 10-cm dishes until 80–90% confluence. For sub-cultivation, cells were washed with prewarmed PBS, harvested using Accutase™ solution, and seeded into the corresponding cell culture plastics depending on the individual assay. THP-1 cells were purchased from LGC Standards (Wesel, Germany) and cultured in RPMI 1640 (Lonza, Basel, Switzerland) containing 5% fetal calf serum (FCS; Biochrom GmbH, Berlin, Germany). Cells were cultured in a standard cell culture incubator (37 °C, 5% CO_2_ humidified atmosphere; Binder GmbH, Tuttlingen, Germany). All culture plastics used for endothelial cell culture were coated with a solution of 0.2% gelatin (Sigma-Aldrich, St. Louis, MO, USA) in PBS before use.

For secretion and gene expression studies, HMVECs were plated into 96-well plates (2 × 10^4^ cells in 0.1 ml per well) or in 6-well plates (1 × 10^6^ cells in 1 ml per well), respectively, and cultured until sub-confluence (80–90% density). Thereafter, cell culture supernatant was replaced by fresh medium, and cells were stimulated with LPS (1 μg/ml), thrombin (1 U/ml), histamine (100 μM) or PMA (50 ng/ml), respectively, in triplicates. Culture medium was used as control. Cells were incubated for 1 h to study secretion into supernatant or for 3 h for gene expression studies. Subsequently, from 96-well plates, cell supernatant was collected and stored at − 80 °C for later analysis. From 6-well plates, supernatant was discarded, and 2 ml of TRIzol™ reagent were added to each well. The adherent cells were removed and lysed using a cell scraper. Cell suspension in TRIzol™ reagent was stored at − 80 °C until extraction of total RNA.

THP-1 cells were plated in 6-well plates (1 × 10^6^ cells in 1 ml per well). After an overnight resting period, cells were stimulated with LPS (0.5, 1 or 2 μg/ml) or with recombinant human TNF-α (0.02, 0.05 or 0.1 μg/ml), respectively, in triplicates. Culture medium was used as control. Cells were incubated for 24 h. Subsequently, the cell suspension was harvested, centrifuged, and 2 ml of TRIzol™ reagent were added to the pellet after discarding the supernatant. The suspension was stored at − 80 °C until extraction of total RNA.

### Animals and murine acute lung injury model

All experiments were performed on female C57BL/6 mice at an age of about 10–12 weeks. Animals were purchased from Charles River (Sulzfeld, Germany) and were housed in individually ventilated, pathogen-free cages with access to water and standard rodent chow (SSNIFF) ad libitum prior to the experiments. The animal protocol of the study was approved by the local committee for animal care (LANUV, Recklinghausen, Germany; protocol no. 84–02.04.2015) and was in accordance with the National Institutes of Health guidelines for the use of live animals (NIH publication No. 85–23, revised 1996).

Intratracheal LPS challenge was performed as described by D’Alessio et al. [14]. In brief, mice were anesthetized with ketamine (120 mg/kg bodyweight (BW)) and xylazin (16 mg/kg BW). LPS (5 μg/g BW, diluted in PBS) was administered intratracheally via a 22-gauge catheter. Control animals received the equivalent volume of PBS (50 μl). According to the protocol, systemic analgesia was performed in all groups via subcutaneous administration of buprenorphine hydrochloride (Temgesic™, Indivior UK Limited, Berkshire, UK; 0.08 mg/kg BW) immediately following ALI induction and every 12 h thereafter.

24 or 72 h following LPS instillation, the animals were sacrificed by CO_2_ asphyxiation, followed by exsanguination. Blood samples were drawn from the vena cava inferior and kept for FACS analysis. Bronchoalveolar lavage (BAL) was performed by puncturing the trachea and lavaging the lung with 2 × 1 ml PBS. BAL fluid was centrifuged for 5 min at 400 x g. BAL supernatant was flash frozen in liquid nitrogen while cells were resuspended in FACS buffer. Lungs were flushed with 10 ml PBS via the right ventricle. Thereafter, spleen and pulmonary tissue were harvested. The right lobe of the lung was divided into two halves, snap frozen in liquid nitrogen and stored at − 80 °C until further use (isolation of proteins and total RNA). Left lung and spleen were homogenized and processed for FACS staining as described in the respective section.

### Detection of ANG2 by ELISA and western blot analysis

ANG2 levels in cell culture supernatant were quantified using a commercially available ELISA kit (R&D Systems (Wiesbaden-Nordenstadt, Germany)) according to the manufacturer’s protocol. Results are given in pg/ml.

Preparation of mouse lung protein extracts from frozen organs using RIPA buffer and western blot analyses were performed according to standard procedures. 50 μg of whole protein extract were used under non-reducing conditions. Equal loading was verified by Ponceau staining. Both the polyclonal rabbit ANG2 antibody (cat. Code 2948, dilution 1:1000) and an anti-rabbit IgG, HRP-linked antibody (cat. Code 7074, dilution 1:2000) were purchased from Cell Signaling Biotechnology (Cambridge, UK). Images were obtained using a digital CCD camera system (Amersham Imager 600, GE Healthcare Life Sciences, Freiburg, Germany).

### Detection of serum albumin in BAL fluid

BAL supernatants were diluted 1:10 in PBS. Albumin content of BAL samples was measured with the Mouse Albumin ELISA Quantitation Set (Bethyl Laboratories, Montgomery, TX, USA) according to the manufacturer’s instructions.

### RNA isolation and quantitative real-time PCR (RT-PCR)

After thawing cells harvested in TRIzol™ reagent, suspension was left at room temperature for 5 min and pipetted up and down for complete cell lysis. The frozen lung samples from murine challenge experiments were homogenized in 4 M guanidinthiocyanat using a stand dispersion unit (Polytron-Kinematica, Lucerne, Switzerland). Tissue debris was removed by centrifugation. Further isolation of total RNA from cells and organs was performed by the TRIzol™ reagent method according to the manufacturer’s instructions using chloroform, isopropanol, and ethanol. The resulting air-dried RNA pellet was resuspended in RNAse-free water, and the concentration was determined spectrophotometrically (NanoDrop, Thermo Fisher Scientific, Waltham, MA, USA). 2 μg of total RNA were reverse transcribed into cDNA using the High Capacity cDNA Reverse Transcription Kit (Applied Biosystems, Weiterstadt, Germany). Following synthesis, cDNA was diluted in RNAse-free water (1:5) and stored at + 4 °C. Gene expression analysis of ANG2, TIE2, IL-8, TNF-α, MMP-9, and VEGF was performed by quantitative RT-PCR with Taqman™ Expression Assays for the respective genes and Taqman™ Gene Expression Master Mix on a ViiA7 device (all Applied Biosystems, Weiterstadt, Germany). Expression was normalized to 18 s ribosomal RNA as house-keeping gene and calculated as fold change expression of the respective control using the delta-delta CT method (RQ, relative quantification).

### FACS analysis of blood, spleen, BAL fluid and lungs

Blood samples were collected from the right ventricle and coagulation was stopped immediately by adding 0.5 M EDTA solution. 200 μl blood were transferred into FACS tubes and red blood cells were lysed after addition of 2 ml red cell lysis buffer (RCB; Thermo Fisher Scientific, Waltham, MA, USA). Reaction was stopped by addition of 1 ml FACS buffer, tubes were centrifuged (4 °C, 400 x g, 5 min), and supernatant was discarded. Cell pellets were washed twice with 2 ml FACS buffer or PBS, respectively.

The BAL cell pellets, resuspended in 500–1000 μl FACS buffer, were filtered through a 100 μm mesh filter, transferred into FACS tubes, and centrifuged for 5 min at 400 x g.

Spleen samples were homogenized, filtered, lysed with 2 ml RCB, and washed with 2 ml FACS buffer (centrifugation at 4 °C, 400 x g, 5 min). Lung tissue was minced and incubated in RPMI (with 10% FCS and 0.1% NaN_3_, Collagenase I 1 mg/ml, DNase II 7 mg/ml) at 37 °C for 60 min. After further homogenization with careful pipetting, the suspension was filtered, centrifuged, lysed with RCB, and washed twice with 1 ml PBS.

Single cell suspensions were incubated with CD16/CD32 antibody (2.4G2, BD Bioscience, Franklin Lakes, NJ, USA) at 4 °C for 5 min to block non-specific binding of immunoglobulin to the Fc receptors. Titrated amounts of the following labelled antibodies from eBioscience (Waltham, Massachusetts, USA), BD Biosciences (San Jose, CA, USA) and BioLegend (San Diego, CA, USA) were used: CD45 (AFS98), F4/80 (BM-8), TIE-2 (TEK4), Gr1 (RB6-8C5), Ly6C (HK1.4), Ly6G (1A8), CD11b (M1/70), together with corresponding isotype controls. Cells were incubated with the antibodies for 15 min at 4 °C in the dark and were washed afterwards. The analysis of the mean fluorescence intensity refers to the fluorescence intensity of each event in average and may be taken as an indicator for the density of an antigen on the investigated cells.

We determined absolute cell numbers by adding fixed numbers of CaliBRITE APC-beads (6 μm) (BD Biosciences, San Jose, CA, USA) before measurement as internal reference, excluding dead cells with LIVE/DEAD Fixable Dead Cell Stain kit (Thermo Fisher Scientific, Waltham, Massachusetts, USA).

We performed flow cytometry on a FACS-Canto II, LSR II, and on a Fortessa (BD Biosciences, San Jose, CA, USA), supported by BD FACSDiva Software version 6.1.2, and analyzed the data with Flow-Jo software version 10.0.5 (TreeStar, Ashland, OR, USA). Immune cells were gated according to size and granularity defined in the forward (FSC) and side light scatter (SSC) plot (see Additional file 1: Figure S1, Supplemental Digital Content). Gating boundaries were set on the basis of control samples at the 24 h time point to minimize data variability (see van der Strate et al. [15]). Cell populations were further characterized based on their live/dead appearance and CD45 expression pattern. The macrophages in lungs and BALF were defined as being CD45^+^, F4/80^+^. The phenotype was differentiated according to the Ly6C and CD11b surface expression, as described by Zaynagetdinov et al. [16]. The neutrophil granulocytes were distinguished from macrophages by being CD45^+^, F4/80^−^, and Ly6G^+^ (Gr1 surface expression level).

### Statistics

Statistical analysis and visualization of data was performed using GraphPad PRISM 5 (La Jolla, CA, USA) and MS Office 2010 (Redmond, WA, USA). Results are presented as mean ± SD. Each experiment was repeated at least three times with identical results. Significance of intergroup differences was tested using Mann-Whitney U test or Kruskal-Wallis one-way analysis of variance followed by Dunn’s post-hoc test, as indicated. The alpha level was set at 5%.

## Results

**Fig. 1:**
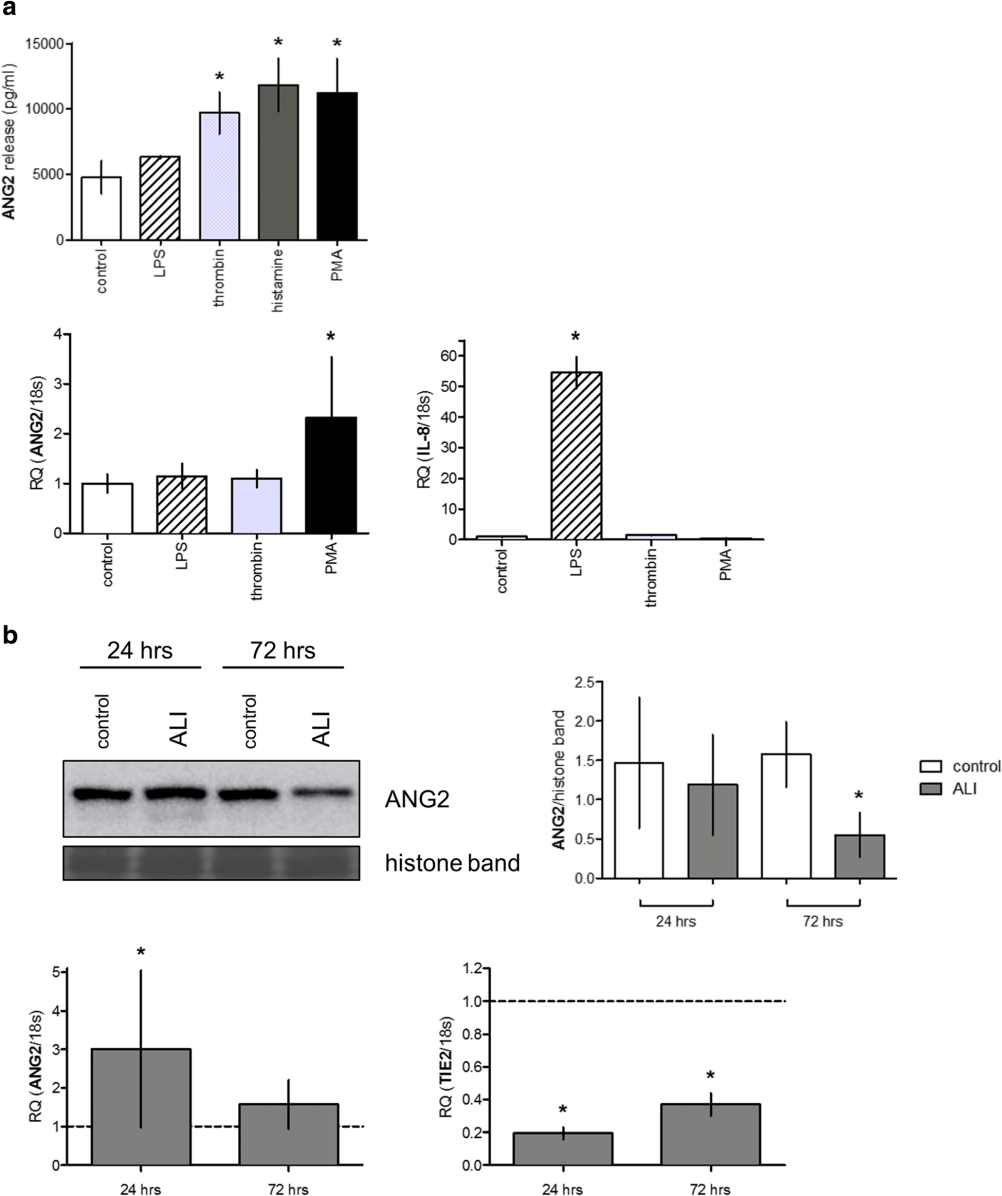
Effect of inflammation on ANG2 expression and release. **a** HMVECs were incubated with LPS (1 μg/ml), thrombin (1 U/ml), histamine (100 μM), or Phorbol 12-myristate 13-acetate (PMA; 50 ng/ml), respectively. The upper panel shows ANG2 release into cell culture supernatant after incubation for 1 h, measured by ELISA. The lower panels show expression of ANG2 (left) and IL-8 (right), respectively, quantified by RT-PCR after 3 h of stimulation. Relative target gene expression was normalized to 18 s RNA and is shown as fold change of expression of the unstimulated control. Mean from biological triplicates ±SD. **b** Female C57BL/6 mice (10–12 weeks old) were challenged by intratracheal instillation of LPS (5 μg/g bodyweight), and 24 or 72 h thereafter, animals were sacrificed and the lungs were harvested. The upper panel shows on the left a representative western blot analysis of lung ANG2 protein content (molecular weight 140 kDa), together with the corresponding histone band (cropped from image of Ponceau staining of the same nitrocellulose membrane). On the right, the results from the densidometric analyses are given, normalized to the histone band (mean ± SD from three blots with identical results). The lower panels show ANG2 and TIE2 expression, quantified by RT-PCR. Relative target gene expression was normalized to 18 s RNA and is shown as fold change of expression of the PBS control (represented by the dashed line). Mean ± SD, *n* = 7. ALI = Acute Lung Injury. Mann-Whitney U test, *p < 0.05 (vs. unstimulated or PBS control, respectively)

HMVECs were incubated with LPS, thrombin, histamine, or PMA, and the effect on ANG2 release and expression was studied using ELISA and RT-PCR. Despite pro-inflammatory response, as evidenced by a significant upregulation of IL-8 expression, incubation of cells with the TLR4 agonist LPS (1 μg/ml) had no direct effect either on ANG2 release from ECs or on its expression (Fig. 1a). However, stimulation with thrombin (1 U/ml), histamine (100 μM) as well as with PMA (50 ng/ml) significantly increased secretion of ANG2 compared to unstimulated control. This was, in case of PMA, accompanied by a weak, yet significant upregulation of its gene expression.

To assess the effect of inflammation on ANG2 activity in vivo, female C57BL/6 mice (10–12 weeks old) were challenged by intratracheal instillation of LPS (5 μg/g BW). After 24 or 72 h, respectively, animals were sacrificed and lung ANG2 expression as well as protein content was analyzed. As evidenced by western blot analysis, LPS challenge reduced ANG2 protein in lung tissue (Fig. 1b). This was maximal 72 h following ALI induction. In contrast, gene expression of ANG2 was significantly enhanced in ALI animals 24 h after LPS instillation compared to control mice, and after 72 h, ANG2 expression began to decline to baseline level. The expression of the ANG2 receptor TIE2 in lung tissue homogenates was significantly reduced to values below 20 to 40% under ALI conditions over the whole observation period.

**Fig. 2:**
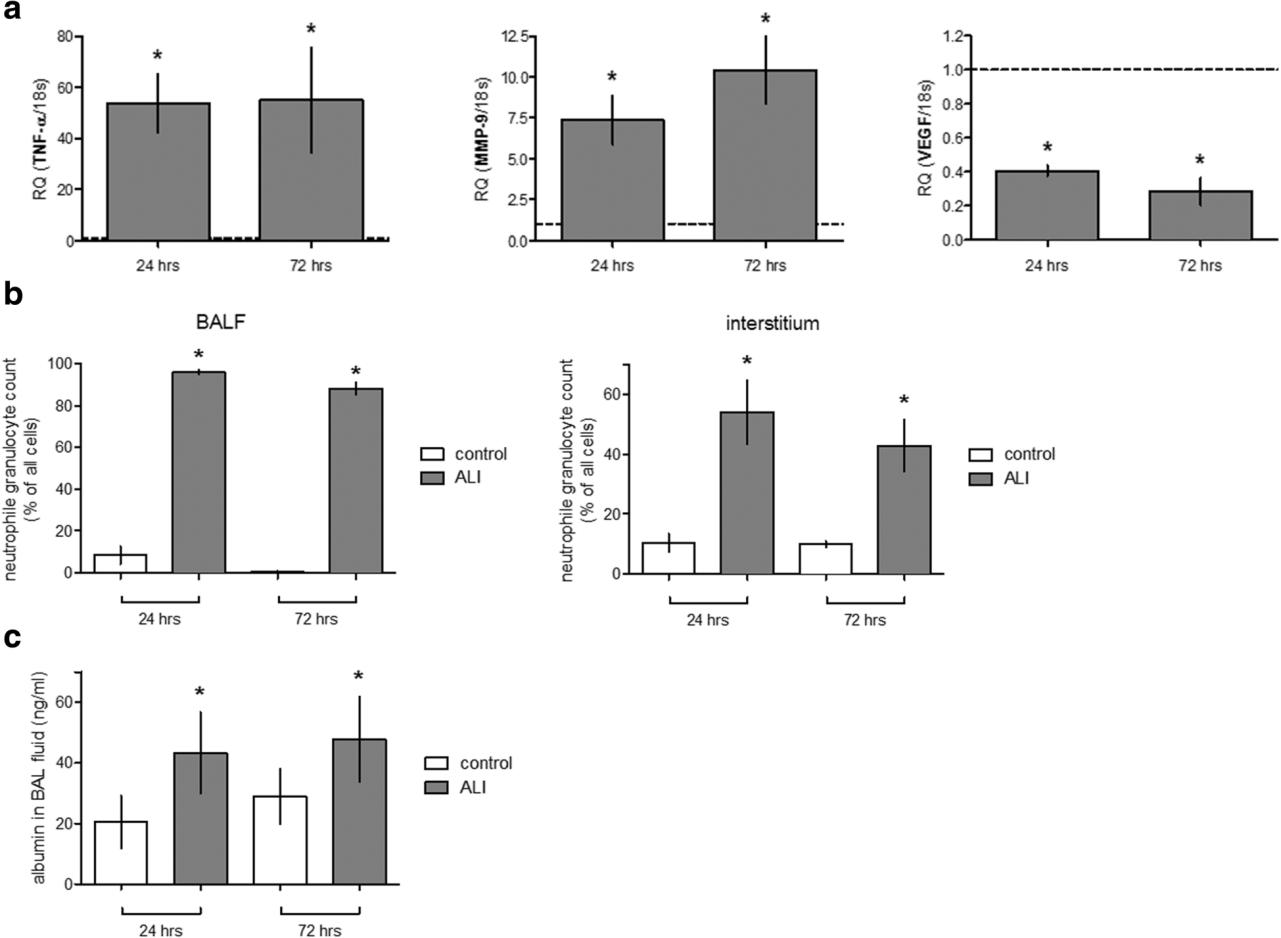
Effect of intratracheal LPS instillation (murine ALI model). Female C57BL/6 mice (10–12 weeks old) were challenged by intratracheal instillation of LPS (5 μg/g bodyweight), and 24 or 72 h thereafter, animals were sacrificed and, after performing bronchoalveolar lavage (BAL), the lungs were harvested. **a** Expression of TNF-α, MMP-9 and VEGF, quantified by RT-PCR. Relative target gene expression was normalized to 18 s RNA and is shown as fold change of expression of the PBS control (represented by the dashed line). **b** FACS analysis of neutrophil granulocyte count in BAL fluid (BALF; left) and in interstitial lung tissue (right). Cell numbers are expressed as percentage of all counted cells. **c** Albumin content was measured in BAL fluid by ELISA. ALI = Acute Lung Injury. Mean ± SD, *n* = 7, Mann-Whitney U test, *p < 0.05 (vs. PBS control)

Tracheal instillation of LPS induced a strong pulmonary pro-inflammatory response. Expression of TNF-α was markedly upregulated, reaching a more than 50fold increase compared to control conditions (Fig. 2a). Expression of the matrix-degrading enzyme MMP-9 showed a significant increase 24 h after ALI induction, further rising after 72 h. The gene expression of VEGF was significantly reduced under ALI conditions. FACS analysis revealed a strong infiltration of neutrophil granulocytes into the lungs of LPS-treated mice, and neutrophils also transmigrated into the alveolar compartment to a large extent, as evidenced by their increased count in BAL fluid (Fig. 2b, Table 1). Systemic response was reflected by significantly elevated neutrophil count in peripheral blood (not shown). In BAL fluid, albumin content was quantified to assess integrity of the alveolo-capillary barrier. Following LPS instillation, endothelial permeability was markedly increased over the whole observation period in comparison to control animals, as evidenced by significantly elevated albumin levels in BAL (Fig. 2c).

**Table 1:** Percentage and absolute frequencies of analyzed leukocyte populations in blood, spleen, bronchoalveolar lavage (BAL) fluid, and lungs

	24 hrs				72 hrs			
	control		ALI		control		ALI	
	%	absolute	%	absolute	%	absolute	%	absolute
Blood								
NG	5.9 (4.8)	2157 (1742)	8.1 (6.1)	2107 (1914)	2.9 (0.4)	1176 (122.1)	14.3 (3.6)	3994 (1407)
Mono	12.5 (9.9)	5635 (5011)	21.7 (10.3)	7763 (6250)	14.2 (2.7)	6755 (1158)	32 (5.9)	13,422 (4165)
TEM	1.4 (0.6)	64.1 (40.8)	2.9 (3.8)	105.6 (113.7)	0.1 (0.1)	6.7 (4)	0.3 (0.4)	35.1 (27.7)
Spleen								
NG	18.8 (15.6)	2890 (3698)	38 (9.9)	7820 (4940)	23.7 (0.3)	12,402 (933.3)	57.2 (9.1)	26,592 (8021)
Mono	6.5 (5.5)	987.9 (1261)	8.5 (3.1)	1520 (526.1)	15.6 (1.5)	8168 (1156)	8.9 (1.1)	4077 (908.4)
TEM	9.5 (10.1)	32.3 (29.4)	2.6 (2.7)	44.9 (53.1)	8.4 (1.2)	676.1 (8.4)	5 (1.5)	202.8 (76.2)
BALF								
NG	8.5 (4.3)	1829 (1724)	95.9 (1.2)	52,005 (21906)	0.7 (0.3)	17 (10.8)	88 (2.9)	37,254 (4478)
MPh	67.3 (3.4)	12,784 (6896)	1.7 (1.4)	957 (841.2)	68.7 (4.1)	1585 (316.7)	5.0 (0.9)	2097 (282)
TEM	23.1 (12.3)	2510 (1108)	13.2 (6.4)	143.4 (141.8)	7.8 (1.5)	121.3 (20.5)	0.6 (0.3)	11.7 (5.2)
Lung								
NG	10.4 (3.1)	7358 (4794)	54.1 (10.6)	65,243 (15855)	9.8 (1.1)	5510 (653.8)	42.9 (8.6)	48,946 (5223)
MPh	40.5 (9.1)	24,571 (10708)	22 (4)	28,503 (12648)	12.1 (1.5)	6817 (866.9)	28.4 (5.6)	34,431 (13593)
TEM	13.8 (7)	3897 (3064)	43.1 (16.7)	12,004 (7267)	5 (1.4)	343.8 (121.2)	8 (4.1)	2646 (1469)

Percentage frequencies of neutrophil granulocytes, monocytes, and macrophages are expressed as percentage of all counted cells, percentage frequencies of TIE2-expressing cells are expressed as percentage of counted monocytes or macrophages, respectively. Values are given as mean (±SD)

*ALI* Acute Lung Injury, *NG* Neutrophil granulocytes, *Mono* Monocytes, *MPh* Macrophages, *BALF* BAL fluid

**Fig. 3:**
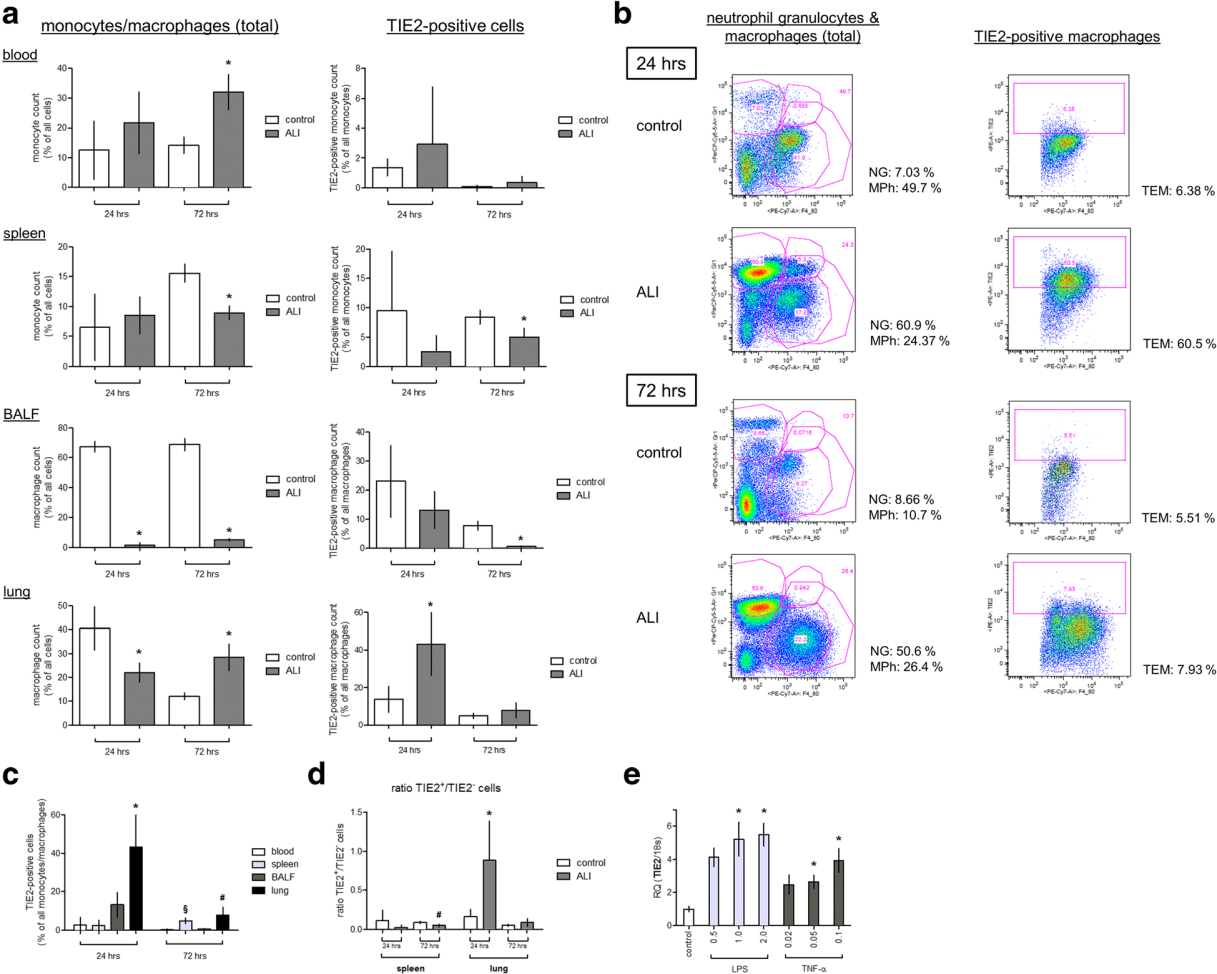
Frequencies of TIE2-negative and -positive monocytes and macrophages during murine ALI. Female C57BL/6 mice (10–12 weeks old) were challenged by intratracheal instillation of LPS (5 μg/g bodyweight), and 24 or 72 h thereafter, animals were sacrificed and frequencies of TIE2-negative and -positive monocytes and macrophages were determined by FACS analysis in blood, spleen, bronchoalveolar lavage (BAL) fluid, and the lungs. **a** Frequencies of monocytes and macrophages (expressed as percentage of all counted cells) are shown on the left side, and fractions of TIE2-expressing cells (expressed as percentage of all monocytes or macrophages, respectively) are shown on the right side. Mean ± SD, *n* = 7, Mann-Whitney U test, * *p* < 0.05 (vs. PBS control). **b** Representative FACS dot plots of lungs from control and ALI animals, respectively, sacrificed 24 or 72 h following ALI induction. Analysis of neutrophil granulocytes (NG) and macrophages (MPh) is shown on the left side, and that of the TIE2-expressing macrophage fraction (TEM) is shown on the right side. **c** Comparison of frequencies of TIE2-expressing cells (expressed as percentage of all monocytes or macrophages, respectively) in all four analyzed compartments. Mean ± SD, *n* = 7, Kruskal-Wallis test with Dunn‘s posthoc analysis, *p < 0.05 (vs. blood, vs. spleen), § *p* < 0.05 (vs. blood), # *p* < 0.05 (vs. blood, vs. BAL). **d** Ratio between relative frequencies of TIE2-positive and -negative monocytes/macrophages cells in spleen (left) and lung tissue (right). Mean ± SD, *n* = 7, Mann-Whitney U test, * *p* < 0.05 (vs. 24 h control, vs. 72 h ALI), # *p* < 0.05 (vs. 72 h control, vs. 24 h ALI). **e** TIE2 expression in THP-1 cells after incubation with LPS or recombinant human TNF-α (concentration as indicated in μg/ml) for 24 h, respectively. Mean from biological triplicates ±SD, Kruskal-Wallis test with Dunn‘s posthoc analysis, * *p* < 0.05 (vs. unstimulated control). ALI = Acute Lung Injury, BALF = BAL fluid

Monocytes and macrophages were quantified in lung, spleen, peripheral blood as well as in BAL using FACS, and macrophages were distinguished from monocytes by positive staining for F4/80. In peripheral blood, ALI induced a significant increase in total monocyte count after 72 h, accompanied by a decline in the spleen (Fig. 3a, Table 1). The TIE2-positive monocyte fraction in peripheral blood likewise tended to be increased under ALI conditions, however, due to low absolute frequencies (below 0.5% of all blood cells), intergroup differences were rather small and did not reach statistical significance. In spleen, TEMs also showed a kinetic similar to those of other monocytes with decreasing count in ALI animals compared to the healthy controls. In BAL, alveolar infiltration of neutrophil granulocytes following instillation of LPS resulted in significant percentage decline of macrophages (MPh), reducing MPh count from nearly 70% (of all cells) in controls to 5% in ALI animals after 72 h. This pattern also involved TEMs. In lung interstitium, during early stage of ALI (within the first 24 h), MPh amount was significantly decreased in comparison to the healthy control mice (22 vs. 40.5% of all cells), whereas 48 h later, the number of MPh in the lungs of ALI mice was elevated (28.4 vs. 12.1% in controls) (Fig. 3a, b, Table 1). In contrast, the amount of TIE2-positive MPh was markedly increased during the first 24 h after ALI induction (43.1 vs. 13.8% of MPh). This resulted in a significantly elevated ratio between TEMs and TIE2-negative MPh in the lungs of ALI animals, whereas the ratio between TEMs and TIE2-negative monocytes was unaffected in spleen (Fig. 3d). Under ALI conditions, TEM numbers in the lungs were the highest, compared to all other analyzed compartments (Fig. 3c). During late stage ALI, TEM proportion in the lungs then fell to 8% of MPh.

To assess TIE2 regulation in monocytic cells under pro-inflammatory conditions in vitro, THP-1 cells were incubated with LPS or human recombinant TNF-α in increasing concentration. This resulted in a dose-dependent, significant upregulation of TIE2 expression compared to the unstimulated control (Fig. 3e).

## Discussion

The main objective of our study was to evaluate if TIE2-expressing monocytes migrate into sites of acute inflammation. By making use of a murine model of Acute Lung Injury, we were able to show that monocytes are recruited from their splenic pool into the animals’ peripheral blood. During early stages of ALI, within the first 24 h, the proportion of TEMs in the lungs increased, while that of other MPh was rather reduced compared to healthy controls. During the further course, the ratio between interstitial TIE2-negative MPh and TEMs then fell back to basal levels. This is the first description of TEM dynamics during acute inflammatory processes.

Since its discovery in the early 1990s, the angiopoietin/TIE receptor system is meanwhile a well-characterized vascular-specific growth factor system. Its causal involvement in acute and chronic inflammatory processes is beyond dispute by now [17]. Especially the effect of a dysbalance between ANG1, mediating vascular quiescence, and ANG2, which blocks ANG1-mediated TIE2 phosphorylation, on endothelial barrier breakdown in vitro as well as in vivo is well described [7–9, 18]. During systemic inflammatory syndromes like ALI/ARDS, SIRS and sepsis, an acute and excessive increase of ANG2 activity results in uncontrolled vascular leakage [7, 9, 19].

For years, it was assumed that ECs are the only cells being capable of functional surface expression of the TIE2 receptor [20]. However, recently, it was discovered that specialized myeloid cells likewise express TIE2, giving them the ability to react to angiopoietins [10]. These TIE2-expressing monocytes and macrophages play a crucial role in the further progression of solid neoplasms. TEMs circulate in low frequencies in the peripheral blood also of healthy individuals, where they have been detected in mice as well as in humans [21, 22]. In our experiments, TEMs accounted for 1–3% of monocytes in the animals’ peripheral blood, which agrees with the literature.

As oxygen supply by diffusion ceases with the further growth of solid malignancies, the tumor rapidly develops central hypoxic regions. In response, an ‘*angiogenic switch*’ induces the overexpression of pro-angiogenic mediators such as ANG2 [23]. The latter is supposed to exert specific chemotactic influence on circulating TEMs, prompting them to migrate into the tumor tissue [11, 22, 24]. There, they support further cancer progression by exerting distinct pro-angiogenic functions [10–12]. In addition, expression of vascular leakage-related mediators and matrix-degrading enzymes such as MMP-9 is increased in TEMs, which facilitates invasive tumor growth and metastasis [12].

ECs are, besides tumor cells, the predominant cell type expressing ANG2 [6]. As inflammation results in the stabilization of hypoxia-inducible factors (HIF), which in turn upregulates ANG2, acute inflammatory conditions (e.g., in ALI) induce ANG2 overexpression [7, 25, 26]. We used tracheal LPS instillation to induce ALI in mice. ECs do not react to direct TLR4 stimulation with upregulation of ANG2 gene expression or release [27]. However, thrombin may be extensively generated in lungs in response to intratracheal LPS administration [28]. It promotes rapid ANG2 release from ECs and, furthermore, activates protein kinase C (PKC), thereby inducing loss of endothelial barrier integrity in various ways [29–31]. Histamine, another known strong inductor of ANG2 release, is also generated in large amounts during LPS-induced ALI [32]. In our experiments, thrombin, histamine as well as PKC activation by PMA resulted in increased ANG2 release from ECs and subsequent upregulation of its expression. In vivo, mice that were challenged with LPS rapidly developed signs of ALI such as significant pulmonary vascular leakage, neutrophil infiltration and intraalveolar migration, as well as extensive upregulation of pro-inflammatory cytokines. Lung ANG2 tissue protein content declined under ALI conditions, reflecting release from cellular storage vesicles into the blood vessel system. The depletion of the intracellular ANG2 pool constitutes an adequate stimulus for the endothelial cell to upregulate ANG2 expression, which was evidenced in ALI animals in comparison to the controls [30]. Although not been demonstrated for LPS-induced direct ALI so far, lung ANG2 upregulation has been reported in models of indirect (remote) ALI [33, 34]. Overall lung TIE2 expression was significantly reduced under ALI conditions in our experiments. This agrees with the recent literature and is, together with increased ANG2 activity, considered to contribute to pulmonary vascular leakage in ALI [7, 35].

In our experiments, induction of ALI induced significant increase in total monocyte count in peripheral blood. Due to low absolute TEM frequencies in blood, their count was not significantly altered. Simultaneous decline of monocytes in spleen suggests recruitment of pooled cells into systemic circulation [36]. In lungs of LPS-challenged mice, during early stage of ALI, number of MPh in interstitium was significantly decreased compared to healthy controls, most obviously due to pulmonary infiltration of neutrophil granulocytes, reducing percentage MPh count. During murine ALI, neutrophil infiltration occurs and peaks early, followed by a delayed, though sustained MPh immigration [37]. This was reflected by increasing MPh count in the further course of ALI in our experiments, while that of neutrophil granulocytes began to fall after 72 h. While in spleen and BALF, the dynamic behavior of the TIE2-positive cell fraction mainly reflects that of the total count of monocytes or macrophages, respectively, FACS analysis of TIE2-expressing macrophages in lung interstitium surprisingly revealed a dynamic different from that of TIE2-negative cells. Early after ALI induction, while count of TIE2-negative MPh decreased, that of TEMs in lung tissue significantly increased. This resulted in percentage TEM numbers being more than three times higher than in lungs of healthy control animals (> 40% of counted MPh), suggesting significant pulmonary TEM infiltration. Of note, TEM numbers in the lungs were the highest, compared to all other analyzed compartments. During the further course, percentage TEM numbers then were significantly reduced compared to early ALI, reaching the level of control mice. Intralesional increase of TEM count is well described, predominantly in solid malignancies, but although specific chemoattraction by ANG2 has been demonstrated in vitro as well as in vivo, the detailed contribution of TEMs from compartments outside a tumor (spleen, peripheral blood) is still uncertain [22, 38]. Venneri et al. could not assess significant differences in TEM frequencies when analyzing peripheral blood samples from healthy donors and cancer patients, and if they may correlate with tumor burden is yet unclear [22, 39, 40]. Our data revealed that the specific dynamic of pulmonary TEMs in ALI was absent in the other analyzed compartments: while the ratio between TIE2-positive and -negative macrophages in lung interstitium was significantly elevated in early stage of ALI and fell to the level of controls during the further course, that ratio between TEM and TIE2-negative monocytes was unaffected in peripheral blood or in the spleen as well, the pool where monocytes are supposed to be recruited from during acute inflammation [36]. This suggests that lung-resident MPh may change their surface characteristics under conditions of ALI rather than the immigration of TEMs from extrapulmonary compartments into the site of inflammation. However, it is a critical limitation of our study that this hypothesis is solely supported by the results from our in vitro experiments. When we incubated human monocytic THP-1 cells with LPS or recombinant TNF-α to assess TIE2 regulation under pro-inflammatory conditions, this elicited a dose-dependent increase in the gene expression of TIE2. That finding may be in line with the results from García et al., who demonstrated enhanced expression of TIE2 in pro-inflammatory polarized macrophages [41]. If TLR activation or the secretion of cytokines in the lungs during ALI contribute to enhanced TIE2 expression in resident macrophages in vivo as well remains to be investigated. Moreover, pulmonary tissue hypoxia which may develop during ALI/ARDS can have an additional impact on leukocyte TIE2 expression [24, 42].

So far, involvement of TEMs has primarily been described in cancerous diseases, either in solid tumor growth or in leukemia [10, 43]. In addition, their occurence was demonstrated under conditions of chronic inflammation such as atherosclerosis, diabetes, or autoimmune disease, in limb ischemia, endometriosis, and in chronic virus hepatitis [44–50]. However, although Murdoch et al. already speculated that TEMs are “likely to be actively recruited to inflamed […] tissues”, our study is the first to describe dynamics of TEMs in a condition of acute inflammation like ALI [24]. But what would be the pathophysiologic significance of acutely increased TEM activity in lung injury or ARDS? Enhanced expression of MMP-9 and VEGF by TEMs could contribute to loss of endothelial barrier function and thus vascular leakage during ALI [12]. However, in our experiments, TEM increase in lungs did not correlate with expression of MMP-9 or VEGF, and, even though increased, due to their relatively low absolute number, a direct effect on vascular leakage seems unlikely. Few authors report on pro-inflammatory properties of ANG2-activated TEMs [41, 47]. However, most likely, they play an immunoregulating role. Besides their pro-angiogenic potential, TEMs possess distinct immunomodulating features. Upon immigration, the stimulation by their specific ligand ANG2 upregulates the expression of IL-10, MRC1 (mannose receptor 1), and CCL17, while that of pro-inflammatory cytokines (TNF-α, IL-6, IL-12, CCL2) is reduced, which is indicative of an alternative, M2-like activation of macrophages [12, 24]. Furthermore, in solid tumors, ANG2-activated TEMs were shown to promote the expansion of regulatory T cells, thus supporting further tumor growth [51]. In fact, recruitment of M2-polarized macrophages into the lungs occurs early during ALI (within 24 h) and is supposed to serve to balance the pro-inflammatory response [37]. Moreover, early, compared to late appearance of IL-10-producing cells in human ALI was shown to influence prognosis [52]. If an increased detection of TEMs, e.g. in BAL fluid, might be of prognostic value remains to be investigated.

## Conclusions

Taken together, our data provide evidence for the first time that the activity of TEMs changes at sites of acute inflammation, as demonstrated in a model of murine ALI. This finding raises several questions, especially on the pathophysiological significance of increased TEM frequencies in such conditions. Although an involvement in immunomodulating processes seems highly likely, further investigations are needed to shed more light on the role of TIE2-expressing monocytes and macrophages in acute inflammatory events.

## Additional file

Additional file 1: **Figure S1.** Gating strategy used during FACS analysis to identify the analyzed leukocytes subsets. Immune cells were gated according to size and granularity defined in the forward (FSC) and side light scatter (SSC) plot. Cell populations were further characterized based on their live/dead appearance and CD45 expression pattern. The neutrophil granulocytes were distinguished from macrophages by being CD45^+^, F4/80^−^, and Ly6G^+^ (Gr1 surface expression level). (TIF 1234 kb)

## Abbreviations

ALI: Acute Lung Injury
ANG: Angiopoietin
ARDS: Acute Respiratory Distress Syndrome
BAL: Bronchoalveolar lavage
BW: Bodyweight
EC: Endothelial cell
ECGM-MV: Endothelial cell basal medium with cell-specific supplements
FACS: Fluorescent Activated Cell Sorting
FCS: Fetal calf serum
FSC: Forward light scatter
HIF: Hypoxia-inducible factor
HMVEC: Human pulmonary microvascular endothelial cell
LPS: Lipopolysaccharide
MPh: Macrophage
PKC: Protein kinase C
PMA: Phorbol 12-myristate 13-acetate
RCB: Red cell lysis buffer
SSC: Side light scatter
TEM: TIE2-expressing monocyte/macrophage
