# Supplementary figures and images for: Experimental murine acute lung injury induces increase of pulmonary TIE2-expressing macrophages
Source: J Inflamm (Lond). 2018 Jun 14;15:12. doi: 10.1186/s12950-018-0188-5 (PMC6001122; doi:10.1186/s12950-018-0188-5)

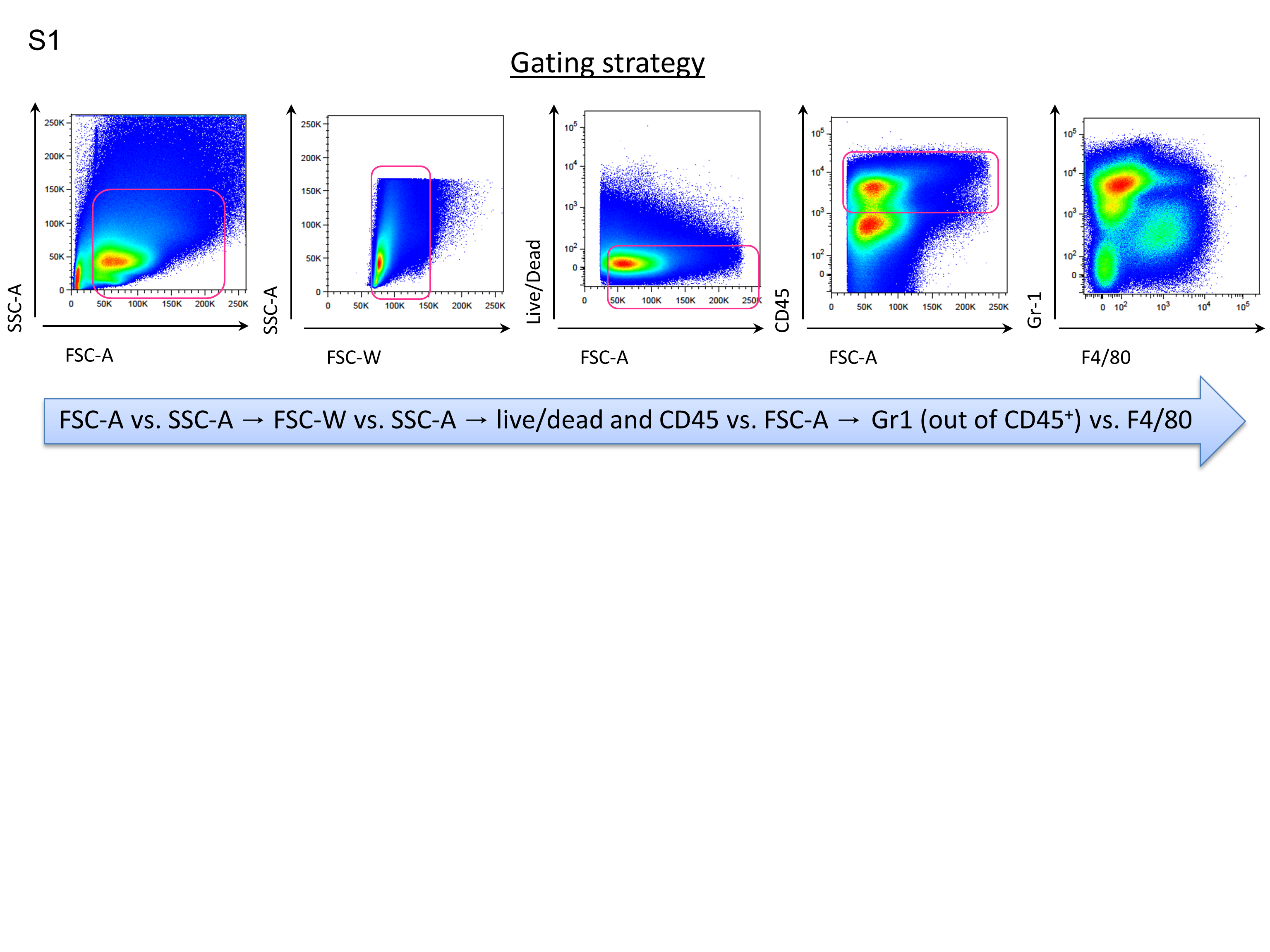

Supplement: Supplementary file 1 — Figure S1. Gating strategy used during FACS analysis to identify the analyzed leukocytes subsets. Immune cells were gated according to size and granularity defined in the forward (FSC) and side light scatter (SSC) plot. Cell populations were further characterized based on their live/dead appearance and CD45 expression pattern. The neutrophil granulocytes were distinguished from macrophages by being CD45+, F4/80−, and Ly6G+ (Gr1 surface expression level). (TIF 1234 kb) [file 12950_2018_188_MOESM1_ESM.tif]
